# Discovery of New Hydrothermal Activity and Chemosynthetic Fauna on the Central Indian Ridge at 18°–20°S

**DOI:** 10.1371/journal.pone.0032965

**Published:** 2012-03-14

**Authors:** Kentaro Nakamura, Hiromi Watanabe, Junichi Miyazaki, Ken Takai, Shinsuke Kawagucci, Takuro Noguchi, Suguru Nemoto, Tomo-o Watsuji, Takuya Matsuzaki, Takazo Shibuya, Kei Okamura, Masashi Mochizuki, Yuji Orihashi, Tamaki Ura, Akira Asada, Daniel Marie, Meera Koonjul, Manvendra Singh, Girish Beedessee, Mitrasen Bhikajee, Kensaku Tamaki

**Affiliations:** 1 Precambrian Ecosystem Laboratory (PEL), Japan Agency for Marine-Earth Science and Technology (JAMSTEC), Yokosuka, Kanagawa, Japan; 2 Marine Biodiversity Research Program, Japan Agency for Marine-Earth Science and Technology (JAMSTEC), Yokosuka, Kanagawa, Japan; 3 Subsurface Geobiology Advanced Research (SUGAR) Project, Japan Agency for Marine-Earth Science and Technology (JAMSTEC), Yokosuka, Kanagawa, Japan; 4 Center for Advanced Marine Core Research, Kochi University, Nankoku, Kochi, Japan; 5 Enoshima Aquarium, Fujisawa, Kanagawa, Japan; 6 Institute of Industrial Science, The University of Tokyo, Meguro, Tokyo, Japan; 7 Earthquake Research Institute, The University of Tokyo, Bunkyo, Tokyo, Japan; 8 Mauritius Oceanography Institute, Quatre-Bornes, Mauritius; 9 Albion Fisheries Research Centre, Petite Rivière, Mauritius; 10 Frontier Research Center for Energy and Resources (FRCER), The University of Tokyo, Bunkyo, Tokyo, Japan; George Mason University, United States of America

## Abstract

Indian Ocean hydrothermal vents are believed to represent a novel biogeographic province, and are host to many novel genera and families of animals, potentially indigenous to Indian Ocean hydrothermal systems. In particular, since its discovery in 2001, much attention has been paid to a so-called ‘scaly-foot’ gastropod because of its unique iron-sulfide-coated dermal sclerites and the chemosynthetic symbioses in its various tissues. Despite increasing interest in the faunal assemblages at Indian Ocean hydrothermal vents, only two hydrothermal vent fields have been investigated in the Indian Ocean. Here we report two newly discovered hydrothermal vent fields, the Dodo and Solitaire fields, which are located in the Central Indian Ridge (CIR) segments 16 and 15, respectively. Chemosynthetic faunal communities at the Dodo field are emaciated in size and composition. In contrast, at the Solitaire field, we observed faunal communities that potentially contained almost all genera found at CIR hydrothermal environments to date, and even identified previously unreported taxa. Moreover, a new morphotype of ‘scaly-foot’ gastropod has been found at the Solitaire field. The newly discovered ‘scaly-foot’ gastropod has similar morphological and anatomical features to the previously reported type that inhabits the Kairei field, and both types of ‘scaly-foot’ gastropods genetically belong to the same species according to analyses of their COI gene and nuclear SSU rRNA gene sequences. However, the new morphotype completely lacks an iron-sulfide coating on the sclerites, which had been believed to be a novel feature restricted to ‘scaly-foot’ gastropods. Our new findings at the two newly discovered hydrothermal vent sites provide important insights into the biodiversity and biogeography of vent-endemic ecosystems in the Indian Ocean.

## Introduction

Hydrothermal systems are known to play key roles in the fostering of high global delta biodiversity in the deep-sea. Over the past 30 years, a number of hydrothermal vent sites have been discovered and investigated, especially in the Pacific and Atlantic Oceans [1]. However, only several hydrothermal fields have been discovered, including quite recent discovery reported from the Southwest Indian Ridge (SWIR) [2], and two of them have so far been investigated in the Indian Ocean [3]–[5]. As a result, in contrast to rapidly accumulating knowledge concerning newly explored hydrothermal systems and associated faunal communities in the Pacific and Atlantic Oceans [6]–[10], information on the biodiversity and biogeography of hydrothermal vent fauna in the Indian Ocean has remained depauperate, even though the deep-sea hydrothermal activity in the Indian Ocean is of great interest in terms of biodiversity and biogeography.

The two previously investigated hydrothermal fields (the Kairei and Edmond fields) are known to host many novel genera and a novel family of animals potentially indigenous to these Indian Ocean hydrothermal systems, in addition to faunal elements common to the Pacific and the Atlantic Oceans [5]. These animal communities lie in a distinct biogeographic province of hydrothermal vent invertebrates, although most of the components also have evolutionary links to their relatives in the Western Pacific and Atlantic hydrothermal environments [4], [5]. Recent statistical work on connections among biogeographical provinces [11] and population connectivities [12] also highlighted similarities between western Pacific and Indian Ocean vent communities. As they pointed out, however, the overall placement of the Indian Ocean hydrothermal vent invertebrate communities with respect to biogeographic province has remained poorly understood because of the paucity of data sets, i.e., only two hydrothermal vent communities found on the Central Indian Ridge (CIR) had been investigated in the Indian Ocean. The accumulation of faunal data from new Indian Ocean vent fields is, thus, required to improve our knowledge of the biodiversity and biogeography of global deep-sea hydrothermal vent fauna.

Among the vent fauna so far reported in the Indian Ocean, much attention has been paid to the ‘scaly-foot’ gastropod, a novel chemosynthetic animal discovered at the Kairei hydrothermal field in the CIR 25°S [5]. The ‘scaly-foot’ gastropod has the distinct features of unusual black-colored iron-sulfide dermal sclerites [13], [14], an iron-sulfide-plated armor shell structure [15], and the unique endo- and epi-symbioses in its enlarged esophageal gland and sclerites, respectively [16]. It is interesting that the existence of the ‘scaly-foot’ gastropod has been reported only in the Indian Ocean and its ecology and morphology and evolutionary history remain mysterious.

In the CIR region around 19°S, an extensive plume survey was conducted in December 2006 and plume signatures of hydrothermally-derived CH_4_, Mn, ^3^He and light transmission anomalies were reported in two regions; on the ridge axial valley of the CIR 18°20′S within the Dodo Great Lava Plain and on the western off-axis slope of the CIR 19°34′S on the Roger Plateau [17]. In October 2009, we conducted seafloor observations using the Human-Occupied Vehicle (HOV) *Shinkai 6500* during the YK 09–13 cruise of the R/V *Yokosuka* in order to locate the seafloor sources of these vent plumes, to characterise the faunal assemblages of their vent fields, and to compare them with the communities known from other CIR vent fields.

## Results and Discussion

### Field observations and fluid chemistry of the new hydrothermal vent sites

**Figure 1:**
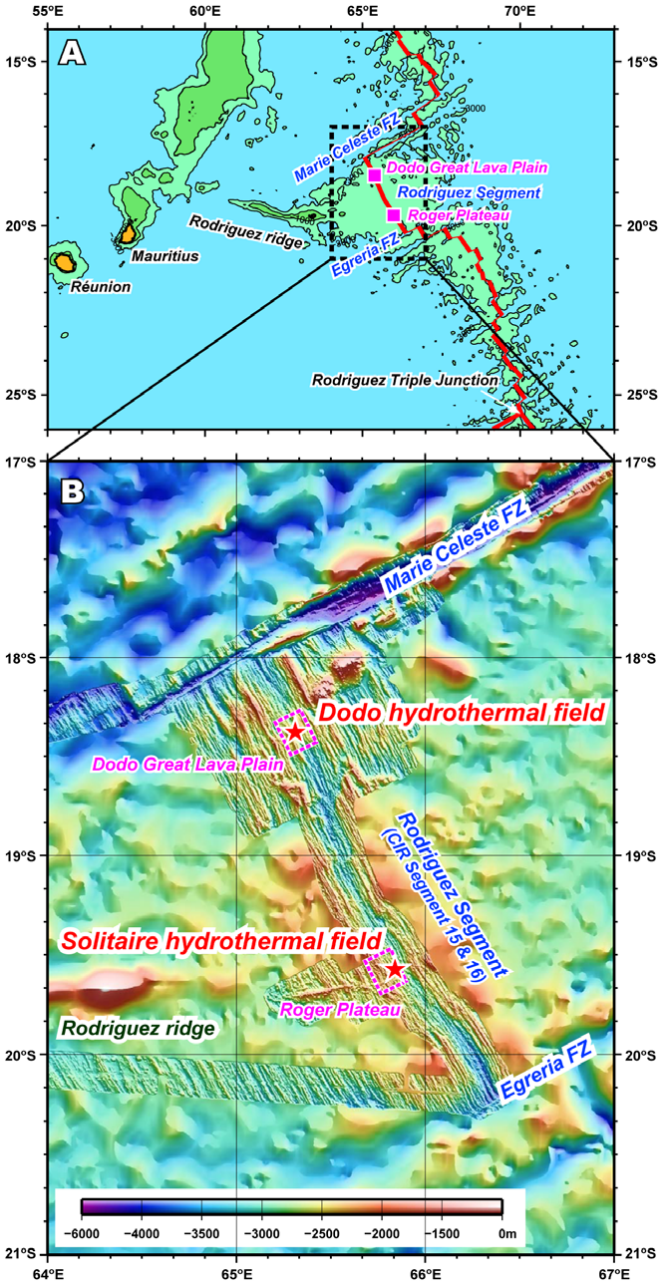
Map of the newly discovered hydrothermal vent fields. (A) Locations of the Dodo Great Lava Plain and Roger Plateau in the Rodriguez Segment (Segment 15 and 16 of the CIR). (B) Locations of the Dodo and Solitaire hydrothermal fields on the Dodo Great Lava Plain and Roger Plateau, respectively.

**Figure 2:**
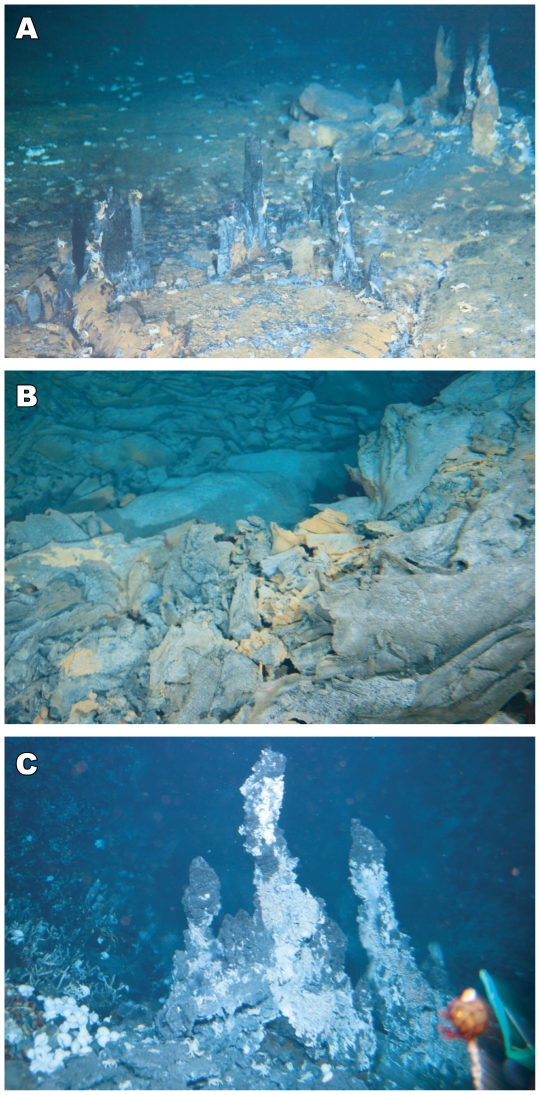
Photographs of black smoker chimneys and associated brownish colored spots. (A) Tsukushi-2 chimneys on basaltic sheet flow lava at the Dodo hydrothermal field. *A. rodriguezensis* is sparsely distributed on the lava. (B) A brownish colored spot in the stained lava area surrounding the Dodo hydrothermal field. (C) Toukon-3 chimneys at the Solitaire hydrothermal field. The chimney walls were partially covered with swarms of *R. kairei*. The animals aggregating around the diffuse fluids are visible in the background to the left of the frame.

During the YK 09–13 cruise, we successfully discovered two new hydrothermal fields, the Dodo and Solitaire fields, on the Central Indian Ridge at 18°–20°S (Movie S1). A new seafloor hydrothermal vent site, the Dodo hydrothermal field (named after an extinct species of bird endemic to Mauritius island) was discovered in the central part of the Dodo Great Lava Plain (18°20.1′S, 65°17.9′E; at a depth of 2745 m) (Figure 1). The setting of the vent field is just on the spreading axis, which is different from the two previously discovered off-axis hydrothermal vent fields (the Kairei and Edmond fields). Hydrothermal fluid emissions were observed within an area of about 15 m in diameter and consisted of many small black smoker chimneys sprouting directly from cracks in the basaltic sheet flow lava without the formation of mounds at their bases (Figure 2A). There were three main chimney sites, named Potsunen, Tsukushi-1, and Tsukushi-2 sites. Most of the chimneys were relatively small, less than 1 m in height. The most vigorous black smoker discharges were observed at the Tsukushi-2 chimney site, at which the highest temperature of hydrothermal fluid (356°C) was sampled. Another two black smoker chimney sites were found in the northern part of the Tsukushi-2 site. In addition to the active black smoker chimneys, several inactive chimneys were observed in the western to southern part of the Tsukushi-2 chimney site. Around the black smoker chimneys we found some chimneys hosting clear, diffuse fluid flows, and diffuse flows issuing directly from cracks and crevices in the basaltic sheet flow lava were also observed. We also found many brownish colored spots with small, collapsed crater-like structures on the basaltic lava surrounding the hydrothermal vent sites (Figure 2B). Some of these colored spots seemed to represent previous black smoker vent sites. Cracks or crevices in sheet flow lava and in the interpillow spaces between pillow lava were also stained by brownish color in this area. The stained area extended more than 50 m in the N-S axis and at least 200 m in E-W axis, with the hydrothermal vent sites being situated in the center of the stained area.

**Figure 3:**
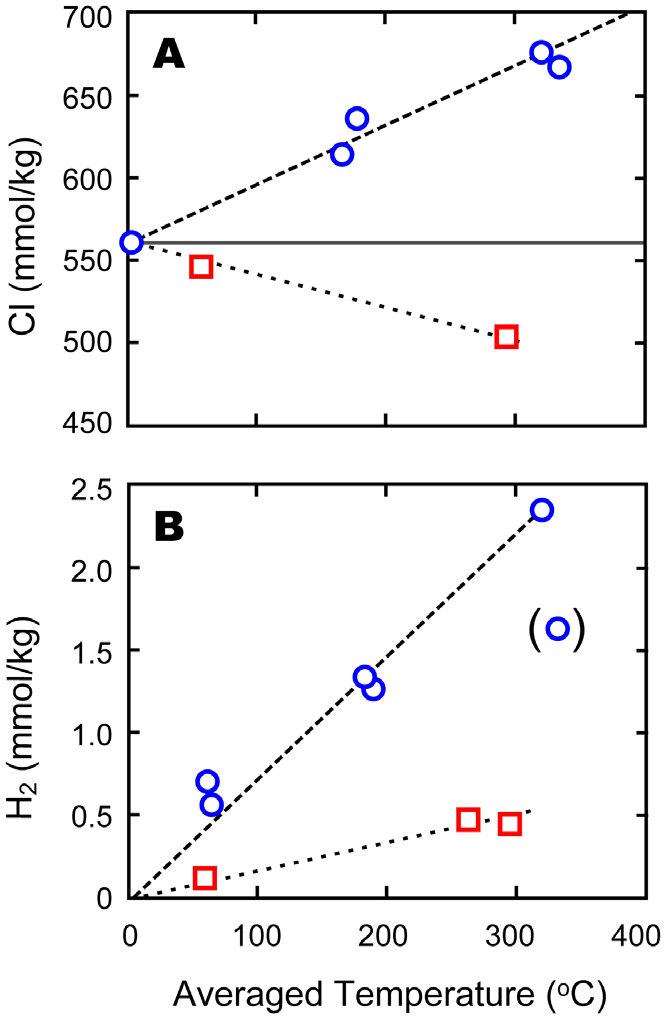
Chemical compositions of hydrothermal fluids from the Dodo and Solitaire hydrothermal fields. (A) Chlorine and (B) H_2_ concentrations are plotted against average fluid temperature during each sampling. Blue open circles with dashed lines and red open squares with dotted lines represent the Dodo and Solitaire fluids, respectively. Gray horizontal line in (A) indicates seawater level (560 mmol/kg).

The highest temperature of the Dodo hydrothermal fluids was measured to be 356°C at the Tsukushi-2 chimney site and the pH of this fluid was 3.2, both of which are comparable with typical Mid Oceanic Ridge (MOR) black smoker fluids. The Cl concentration in the hydrothermal fluids was significantly (∼20%) enriched from that in seawater (Figure 3A), suggesting subseafloor phase-separation and brine-phase enrichment. The concentrations of H_2_ in the Dodo hydrothermal fluids (>2 mmol/L) were notably high (Figure 3B), while those of CH_4_ (∼0.02 mmol/kg) and CO_2_ (∼4 mmol/kg) were comparable with typical MOR hydrothermal fluids [18]. Such high concentrations of H_2_ in the hydrothermal fluid are quite atypical for basalt-hosted hydrothermal systems, with the notable exception of the extremely vapor-rich hydrothermal fluids reported from the East Pacific Rise [19]. The brine-enriched chemistry and low CH_4_ concentration suggest, however, that gas condensation through phase separation [20] and thermal decomposition of organic matter [21] were both little involved.

Another seafloor hydrothermal vent site, the Solitaire hydrothermal field (named after an extinct species of bird endemic to Rodriguez island) was discovered on the western flank of the rift valley of CIR segment 15 (19°33.413S, 65°50.888E; at a depth of 2606 m), eastern edge of the Roger plateau (Figure 1), by two Deep-tow camera surveys and one HOV dive. The Solitaire field setting was on talus at the base of a NNW-SSE trending steep cliff, regarded to be a fault scarp. The area of the hydrothermal emissions was approximately 50 m by 50 m, which is much larger than the Dodo hydrothermal field and comparable with the Kairei and Edmond hydrothermal fields. At the Solitaire hydrothermal field we found three major chimney sites, named the Toukon-3, Tenkoji, and Liger chimney sites. The observed chimneys were less than 5 m in height and were situated on talus with the formation of small mound at their feet, although most off-axis hydrothermal fields (e.g., the Kairei and Edmond hydrothermal fields) are known to have big, complex chimney structures with large massive sulfide mounds at their bases [22]. Several black smoker discharges were observed at the top of the Toukon-3 chimneys (the highest temperature of hydrothermal fluid was 296°C) (Figure 2C), while most of the hydrothermal emissions in the Solitaire field were clear fluids. The Solitaire field is characterized by numerous diffuse flows issuing from the talus throughout the hydrothermal field. Compared with other CIR deep-sea hydrothermal fields, including the Dodo field, the extensive occurrence of diffuse flows is atypical. It seems very likely that the extensive occurrence of thick and highly permeable talus provides a broad range of mixing zones in the subseafloor environments between the upwelling high temperature hydrothermal fluids and the infiltrated seawater. This would also provide a broad spectrum of physical and chemical habitat conditions for subseafloor and seafloor microbial and macrofaunal communities.

The maximum temperature of the Solitaire fluids (296°C) was measured at the Toukon-3 chimney site. The fluids had chlorinities about 10% lower than ambient seawater, indicating subseafloor phase-separation and segregation of a vapor-rich phase for the obtained fluid (Figure 3B). Solitaire fluids are also characterized by higher pH (4.8 measured at 25°C and 1 atm) compared to the other CIR hydrothermal vent fluids [23], [24]. H_2_ concentrations (up to 0.46 mmol/L) are much lower than in the Dodo fluids, but generally comparable with typical MOR hydrothermal fluids such as the Edmond fluids [23], [24]. CH_4_ (∼0.05 mmol/kg) and CO_2_ (∼8 mmol/kg) concentrations are also within the typical range of MOR hydrothermal fluids [18].

Solitaire and Dodo fluids are also characterized by high K/Cl ratios, both of which are among the highest reported in MOR hydrothermal fluids [23]. The basement Mid Oceanic Ridge Basalt (MORB) at the Dodo and Solitaire hydrothermal fields is known to be enriched in alkaline-elements due to the influence of plume components derived from the Réunion hot spot [25]. It is, therefore, very likely that the high K/Cl ratios of the Dodo and Solitaire fluids reflect the distinct chemistry of the plume-related MORB at the base of the hydrothermal fields.

### Hydrothermal vent faunas

**Figure 4:**
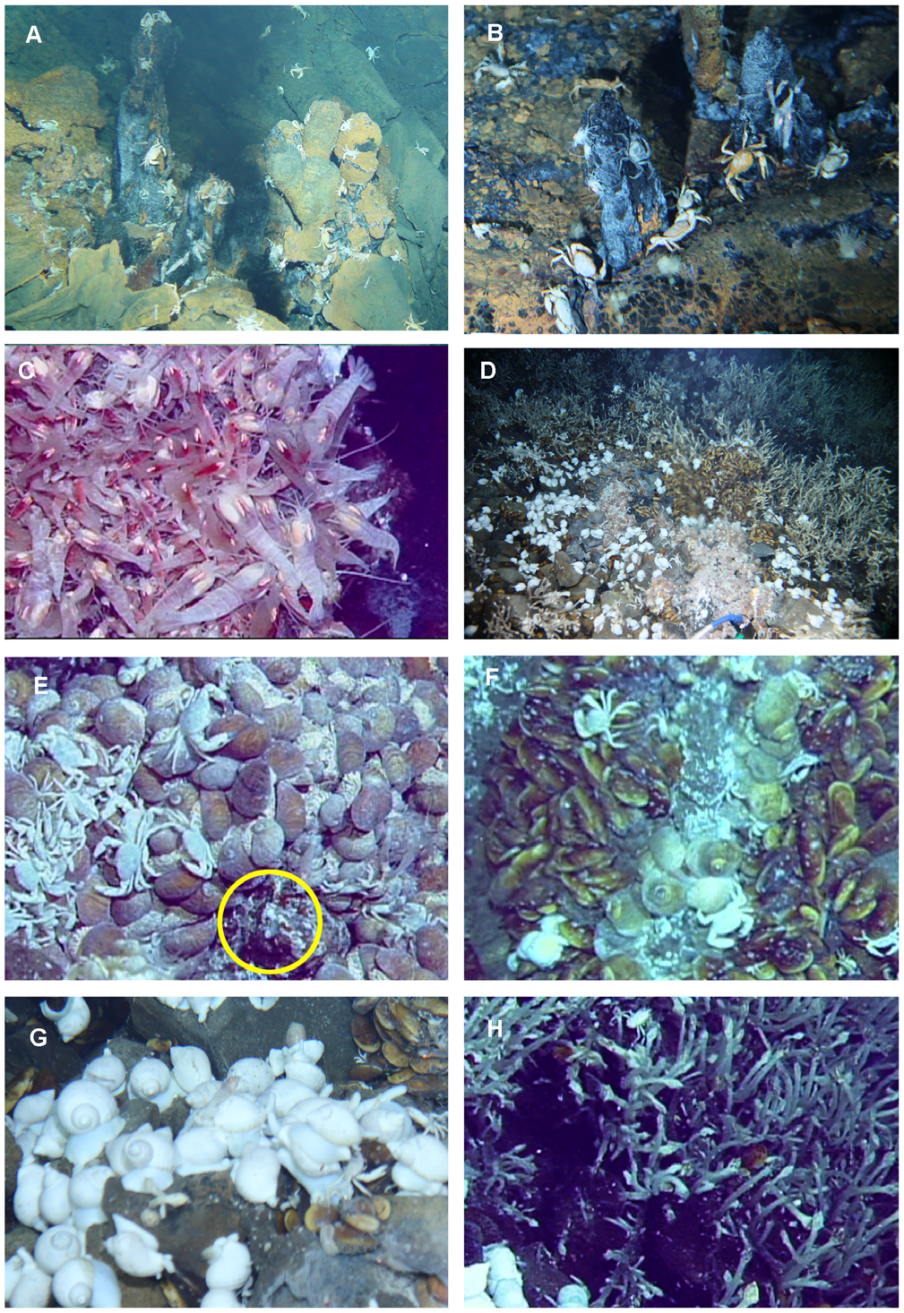
Invertebrates found at the two newly-discovered hydrothermal fields on the Central Indian Ridge. (A) *A. rodriguezensis* and alvinocaridid shrimps (*R. kairei* or its relatives) around a black smoker at the Dodo field. (B) *A. rodoriguezensis*, alvinocaridid shrimps, and sea anemones around small chimneys on the stained seafloor at the Dodo field. (C) Dense aggregations of alvinocaridid shrimps (predominantly *R. kairei* with some *Mirocaris indica*) on the chimneys in the Solitaire field. (D) Overview of faunal assemblage around the diffuse flow vent in the Solitaire field. (E) Aggregations of the new morphotype of ‘scaly-foot’ gastropod and a colony of alvinellid polychaetes (yellow circle) at the Solitaire field. (F) *Alviniconcha* gastropods surrounded by *Bathymodiolus* mussels at the Solitaire field. (G) *Phymorhynchus* gastropods and (H) *Neolepas* barnacles in the outermost part of the zone around diffuse vent fluids at the Solitaire field.

At the Dodo field, only a few hydrothermal vent animals were observed and sampled (Table 1). The bythograeid crab *Austinograea rodriguezensis* was distributed over a relatively wide area of brownish stained lava around the vent fields (Figure 2A). Its population was most abundant adjacent to the black smoker chimneys (Figure 4A). The second dominant species was the alvinocaridid shrimp *Rimicaris kairei*. The shrimp was patchily distributed on the surface of the black smoker chimneys (Figure 4B) and on the lava adjacent to the hydrothermal fluid flows. At the Kairei and Edmond fields in the CIR, the alvinocaridid shrimps are dominant and thousands of individuals cover the hydrothermal vent chimneys and mound surfaces [4], [5]. By contrast, only 10–20 individuals were observed at the Dodo hydrothermal field. As well as these crab and shrimp species, a sea anemone (*Marianactis* sp.), and some small gastropods (lepetodrilid limpets and provannid snails) were sampled. Although the small-scale distribution of the gastropods could not be characterized during the submersible observations because of their small size, anemones were widely distributed throughout the stained area. With increasing distance from the center of hydrothermal activity, the size of individuals became smaller. As far as we observed, no *Alviniconcha* gastropods, ‘scaly-foot’ gastropods or mussels were present at the Dodo hydrothermal field.

**Table 1:** List of the observed and/or sampled animals in the Dodo and Solitaire fields.

Vent Field	Phylum	Species
Dodo Field	Cnidaria	*Marianactis* sp.
	Arthropoda	*A. rodoriguezensis*
		*R. kairei*
Solitaire Field	Cnidaria	*Marianactis* sp.
	Arthropoda	*A. rodoriguezensis*
		*R. kairei*
		*Chorocaris* sp.
		*Mirocaris indica*
		*Munidopsis* sp.
		*Leucolepas* sp.
		*Eochionelasmus* sp.
	Mollusca	*Bathymodiolus* sp.
		*Lepetodrilus* sp.
		*Alviniconcha* sp.
		*Desbruyeresia* sp.
		*Phymorhynchus* sp.
		scaly-foot' gastropod
	Annelida	allvinellid gen et sp.
		Polynoidae gen et sp.
	Echinodermata	Apodacean gen et sp.
	Cordata	Macrouridae gen et sp.

Although the exact cause of the distinct size and composition of the faunal community observed at the Dodo vent site is still uncertain, it is noteworthy that this vent site is characterized by significantly smaller chimneys standing directly on fresh sheet flow lava without any sulfide mound structures or sediment cover. These observations clearly suggest that the start of hydrothermal fluid venting at the vents is quite recent and duration of the hydrothermal activity is fairly short. This could be a reason for the small size of the faunal community.

As opposed to the Dodo field, the biomass and observed taxonomical richness of the faunal communities were high at the Solitaire field (Table 1). Since only one HOV dive was conducted at the Solitaire field, the seafloor observations and sampling of the vent fauna were not enough to accurately describe the communities. Dense populations of *Rimicaris kairei* mixed with *Mirocaris indica* were observed on the chimney structures hosting high temperature fluids (Figure 4C). Near the chimney structures, a diverse fauna with a clear zonation pattern was observed; (1) scaly-foot and *Alviniconcha* gastropods and alvinellid polychaetes were distributed just adjacent to numerous diffuse flows (50–100°C, Figure 4D, E), (2) *Bathymodiolus* mussels with numerous lepetodrillid limpets attached, inhabited mid-distances from the chimneys, and (3) *Phymorhynchus* gastropods, scale worms and at least two species of barnacles (*Neolepas* sp. and *Eochionellasmus* sp.) inhabited areas further from the chimneys. *A. rodriguezensis* and an undescribed species of *Chorocaris* shrimp occurred around the gastropod, and *Bathymodiolus* zones, while only a few *R. kairei* were observed in this area. Macrourid fish, *Marianactis* anemones, *Munidopsis* squat lobsters and apodacean holothuroids were occasionally observed outside of the area inhabited by the diffuse-vent communities.

The distribution of the scaly-foot and *Alviniconcha* gastropods at the Solitaire fields differed from that at the Kairei field. The two gastropods were distributed near high-temperature diffuse fluids issuing from the chimney structures at the Solitaire field. In contrast, both gastropods coexist adjacent to low temperature diffuse flows from the wall of a single large chimney (named Monju) at the Kairei field [4], [5]. Because both of these gastropods harbor symbiotic bacteria and they require specific environments, differences in the hydrothermal fluid chemistry between the Solitaire and Kairei fields could be an important factor contributing to the different distribution of these gastropods.

Another notable discovery at the Solitaire field was the presence of alvinellid polychaetes. This is the first report of such at an Indian Ocean hydrothermal vent field. The distribution of alvinellid polychaetes suggests a faunal connection between the Pacific and Indian Oceans (Table S1), although the observed alvinellid population was small compared to those at other hydrothermal vent fields in other biogeographical provinces in the eastern and western Pacific. Moreover, as the scaly-foot gastropod must belong to the Peltospiridae, which is a group mostly distributed in eastern Pacific vent fields, the presence of the scaly-foot gastropod at two of four CIR vent fields also suggests relationships between Indian Ocean and eastern Pacific hydrothermal vent fauna (Table S1).

### A new morphotype of ‘scaly-foot’ gastropod without iron-sulfide dermal sclerites

**Figure 5:**
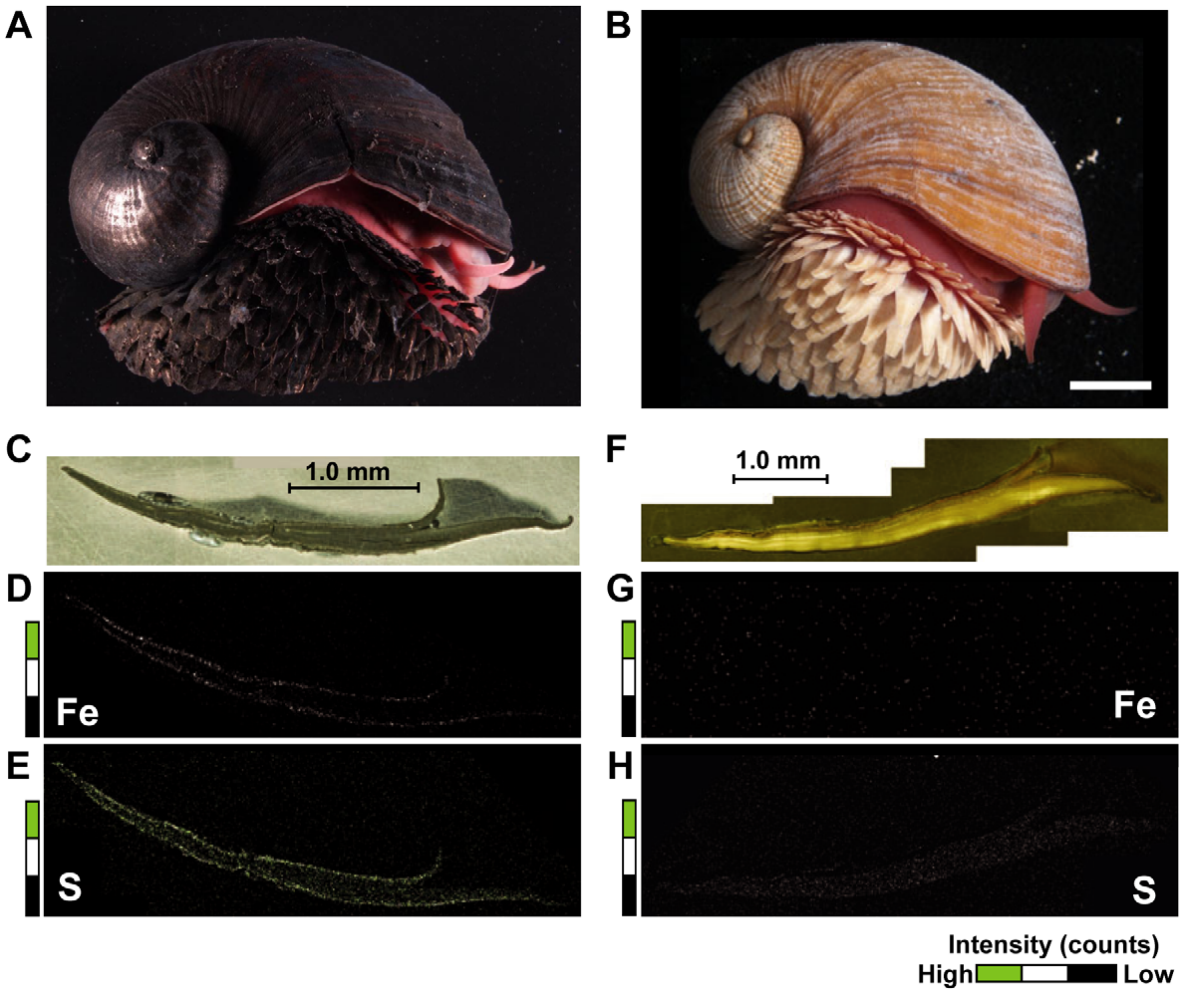
Photographs of macromorphology and of microscopic and SEM-EDS analyses of sclerites from the two morphotypes of ‘scaly-foot’ gastropods. (A), (B) Photographs of the two morphotypes of ‘scaly-foot’ gastropods. Bar indicates 1 cm. (C), (F) Optical micrographs of the polished surface of the sclerite sections. (D), (G) Elemental mapping of iron in the same sections by SEM-EDS. (E), (H) Elemental mapping of sulfur by SEM-EDS. The black-white-green colors indicate the intensity of each element.

**Figure 6:**
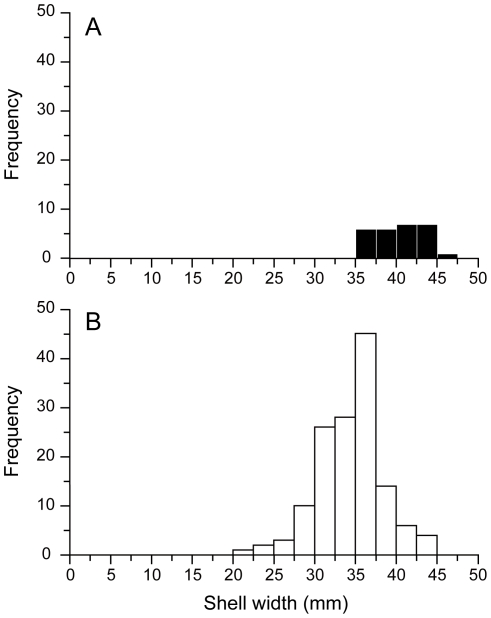
Size distributions of ‘scaly-foot’ gastropods at the Kairei (A) and Solitaire (B) fields. The average sizes of the Kairei and Solitaire populations were 37.87 mm and 31.95 mm, respectively.

**Figure 7:**
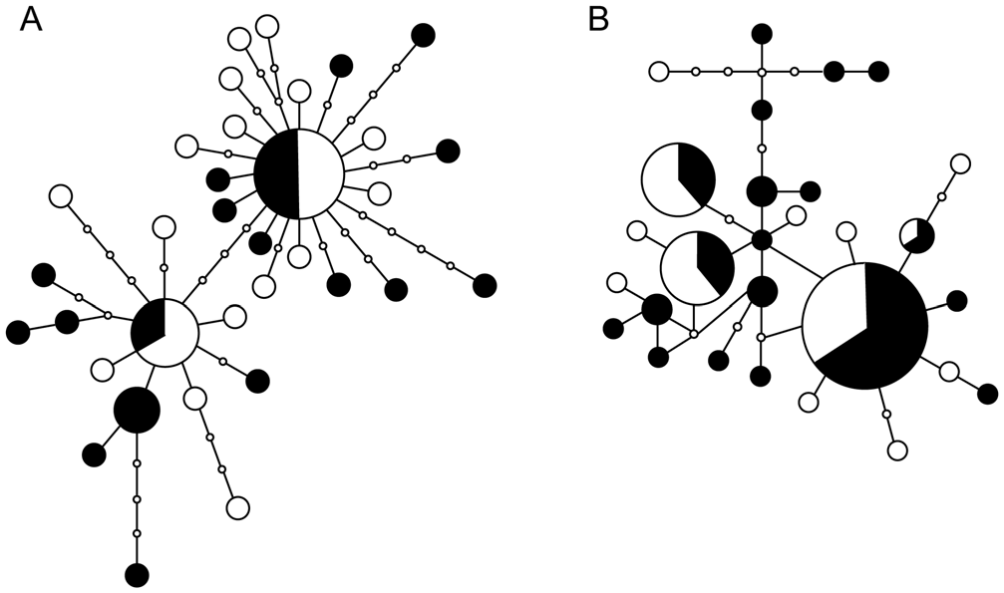
Parsimonious networks of the ‘scaly-foot’ gastropods at the Solitaire and Kairei fields, based on sequences of nuclear SSU rRNA gene (A) and mitochondrial COI gene (B). White circles indicate the haplotypes from the Solitaire field and black circles indicate those from the Kairei field.

The new morphotype had a brown shell and cream-colored sclerites, in contrast to the metallic black shell and sclerites of the type previously known from the Kairei field (Figure 5A, B). Although the ‘scaly-foot’ gastropod from the Solitaire site was slightly smaller in average size (Wilcoxon-Mann-Whitney test, U = 2315, z = 1.919, P<0.05; Figure 6), the general morphological and anatomical features other than the colors of the sclerites and shell were very similar between the Kairei and Solitaire types. Phylogenetic and haplotype analyses based on the sequences of the nuclear small subunit (SSU) rRNA gene and the mitochondrial cytochrome *c* oxidase subunit I (COI) gene clearly demonstrated that both color morphotypes of ‘scaly-foot’ gastropods were genetically indistinguishable and should be classified as the same species (Figure 7).

Elemental mapping of polished sections of sclerites from the two morphotypes was conducted by Scanning Electron Microscopy-Energy Dispersive X-ray Spectroscopy (SEM-EDS) analyses. Both types of sclerites contained carbon, nitrogen and oxygen as the potential protein signatures. The sclerite from the Kairei morphotype also exhibited a clear condensation of iron (Fe) and sulfur (S) elements specifically in the black outer parts (Figure 5C, D, E), indicating the presence of a thick iron-sulfide mineral (e.g., greigite and pyrite) coating on the sclerite as previously reported [13], [14]. In striking contrast, no specific condensation of Fe and S was identified in the sclerite of the Solitaire morphotype (Figure 5F, G, H). This result suggests that the two morphotypes of ‘scaly-foot’ gastropod have different mineralization capabilities. Moreover, it has recently become evident that the Kairei morphotype has unique protection mechanisms associated with the iron-sulfide-plated shell [15]. The different colors of the shells could, thus, also affect the protection mechanism.

In order to examine how the different mineralization mechanisms affect the potential physiological role of the sclerites, we compared the mechanical properties of sclerites from the two morphotypes (Table 2). It has been postulated that the iron-sulfide-mineralization of sclerites in the Kairei morphotype serves to harden the sclerites for protection from predation [14]. Surprisingly, the non-iron-sulfide-mineralized sclerite from the Solitaire morphotype showed greater mechanical strength of the whole structure than did that from the Kairei morphotype (Table 2). This result implies that the iron-sulfide-mineralization does not necessarily improve the mechanical strength of the sclerites for protection from predators, which once again makes the physiological role of iron-sulfide-mineralization unclear.

**Table 2:** Comparison of stress of both sclerites obtained from three-point bending test.

	White sclerite	Black sclerite
Stress (MPa)	12.06±1.06	6.54±3.07

**Figure 8:**
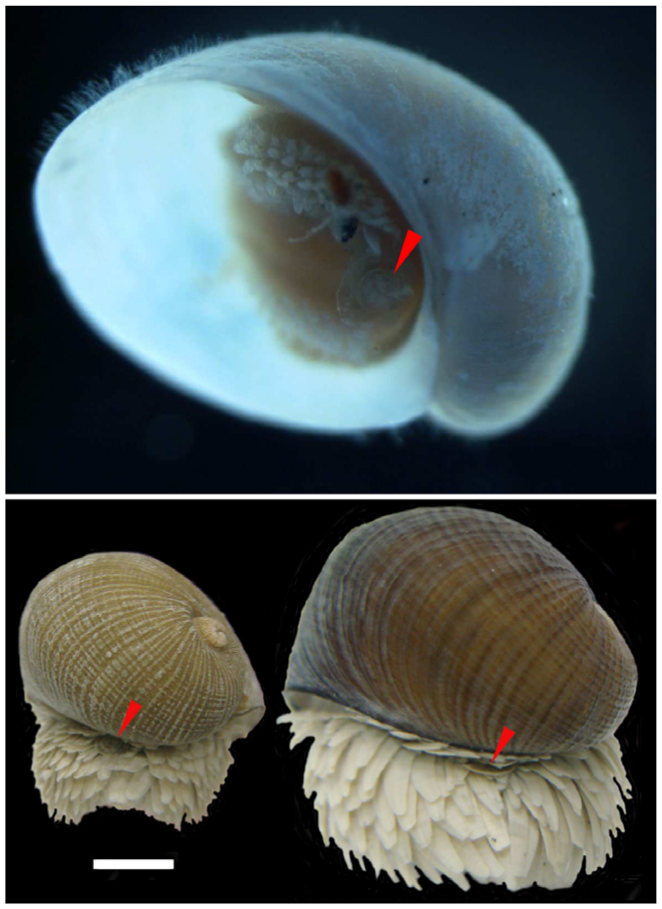
Opercular plates of the ‘scaly-foot’ gastropod. (A) Ventral view of juvenile of ‘scaly-foot’ gastropod with operculum indicated by red arrowheads. Shell length is about 2 mm. (B) Opercula of adult ‘scaly-foot’ gastropods indicated by red arrowheads. The scale bar represents 5 mm.

**Figure 9:**
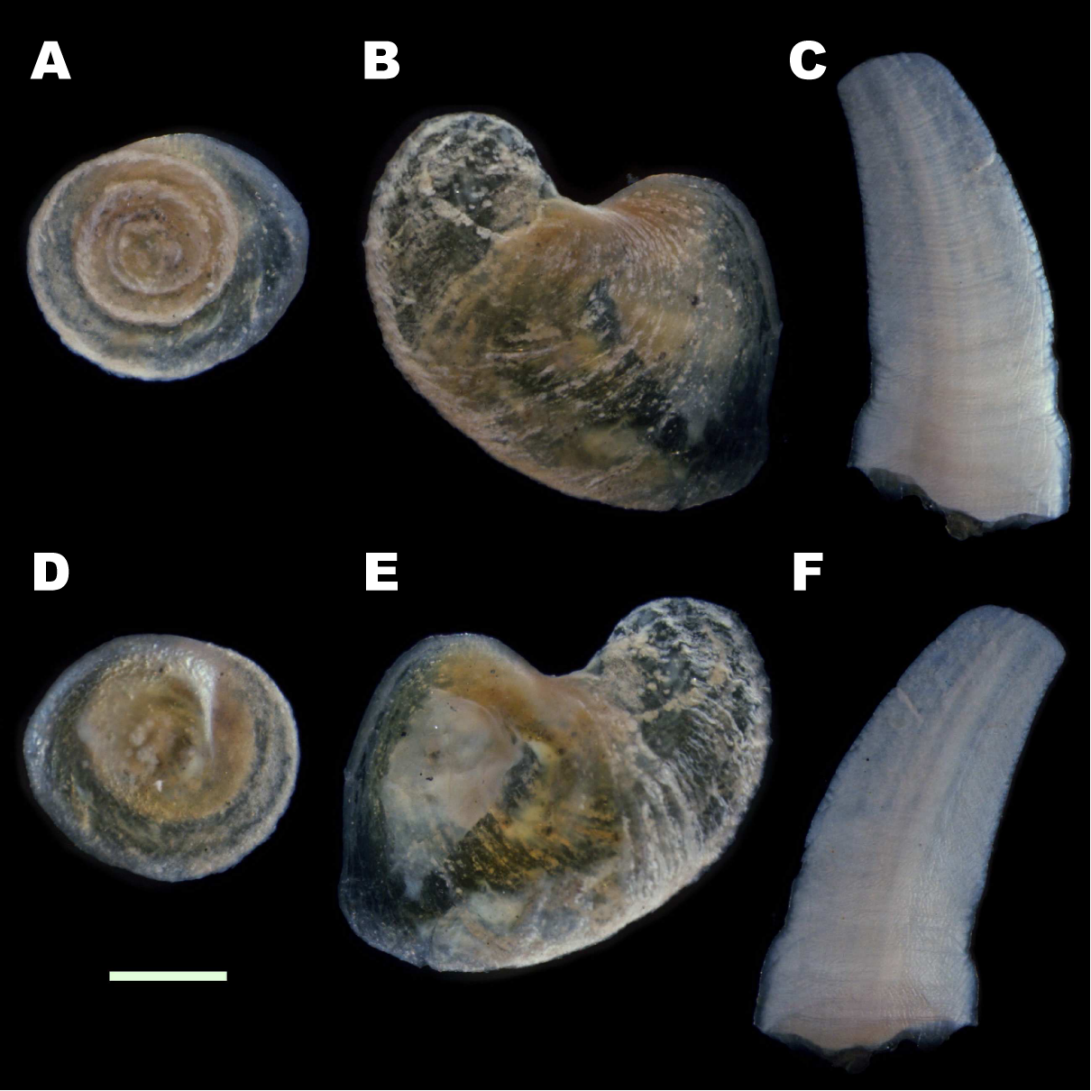
Morphological differences in the vestigial opercula of ‘scaly-foot’ gastropods. (A) Dorsal surface of the operculum of a juvenile individual. (B) Dorsal surface of the operculum of an adult individual. (C) Outer surface of the sclerite of an adult individual. (D) Ventral surface of the operculum of a juvenile individual. (E) Ventral surface of an adult individual. (F) Inner surface of the sclerite of an adult individual. Scale bar indicates 1 mm.

It has also been believed that the characteristics of adult ‘scaly-foot’ gastropod are the absence of an operculum with well-developed sclerites covering the foot, although the origin of these sclerites remains uncertain [13], [26]. During the present investigation, a juvenile ‘scaly-foot’ gastropod with a vestigial opercular plate and sparse sclerites on the lateral sides was collected (Figure 8A). Interestingly, about a half (18 of 34 individuals) of the adult ‘scaly-foot’ gastropods in the Solitaire site also had a vestigial opercular plate among the sclerites at the rear of the animal (Figure 8B), and the morphology of the operculum changed during growth, from a rounded shape in juveniles to a curved shape in adults (Figure 9). It seems likely that the sclerites gradually proliferate and fully cover the whole foot for protection, while the operculum loses its protective function as the animal grows. The present observation revealed that opercular plates are not absent in the ‘scaly-foot’ gastropods even in the adult stage, and, based on the morphological similarities, that they may have some relationships to the sclerites.

### Future prospects

Seafloor reconnaissance by the HOV Shinkai6500 successfully located two new hydrothermal fields (the Dodo and the Solitaire fields) on the CIR at 18°–20°S. The preliminary characterization of the geological settings, the fluid emission patterns, the fluid chemistry and the associated faunal communities greatly extended our knowledge of geochemical variations of hydrothermal activity and of the biodiversity and biogeography of the hydrothermal vent-associated fauna in the Indian Ocean.

The unusually high H_2_ concentrations in the slightly brine-enriched hydrothermal fluids at the Dodo field provides several challenging questions to resolve: what kinds of (hydro)geological and hydrothermal processes in the basaltic lava-hosted (non-ultramafic-associated) Dodo field contribute to the enrichment of H_2_ in the hydrothermal fluids, and how does the unique hydrothermal fluid chemistry affect the formation and function of seafloor and subseafloor microbial communities. Another important question arising from the discovery of the Dodo field is that of why the vent field is comparatively depauperate in fauna, and why is it also clearly distinct in composition from the other CIR vent fields (dominated by bythograeid crabs, rather than alvinocaridid shrimp, with no or few mussels and gastropods) (Table S1).

On the other hand, future investigations of the Solitaire field, which hosts rich and diverse megafaunal communities (Table S1), could result in further discoveries of as-yet unseen and unsampled Indian Ocean vent fauna. This could improve our knowledge of biodiversity of the Indian Ocean vent faunas as well as their biological connections among hydrothermal vent communities in the Indian, Atlantic, and Pacific Oceans. In addition, it is now evident that the novel biomineralization of iron-sulfide in the sclerites and shell of ‘scaly-foot’ gastropods is not species-specific but may be an acquired or lost phenotype during species propagation and adaptation to different Indian Ocean deep-sea hydrothermal systems. Not only questions regarding this mineralization, but also the symbioses in the different morpho- and eco-types of ‘scaly-foot’ gastropods are now under extensive investigation.

It is clear that detailed physiological, genetic, and trophic characterizations of hydrothermal vent-endemic faunas in the new hydrothermal fields in the CIR, as well as quite recently discovered ones in the SWIR [2], will shed light on the evolutionary and propagatory links of each faunal component among the Indian Ocean and between the mid-ocean-ridge hydrothermal systems.

## Materials and Methods

### Hydrothermal fluid sampling and chemistry

Hydrothermal fluid samples were collected at both of the two newly discovered hydrothermal fields (the Dodo and Solitaire fields) on the Central Indian Ridge at 18°–20°S (described below) by using a gas-tight fluid sampler WHATS II [27]. Because the sampling sites are within the EEZ (Exclusive Economic Zone) of the Republic of Mauritius, we collected the samples with permission from the Mauritius Prime Minister's Office. Fluid temperatures were monitored simultaneously during fluid sampling using a temperature probe at the top of the fluid inlet tube. The pH and chloride ion concentrations of fluids were determined onboard using a pH meter with a combined glass electrode (Metrohm, 794 Basic Titrino) at room temperature and the Mohr titration method, respectively. In addition, the dissolved gas components in the hydrothermal fluids were extracted and their concentrations were determined onboard. The fluids in gas-tight sampler bottles were opened to a vacuum line to recover the gas components. After degassing, approx. 50 cc of the gas phase was subsampled. Total gas contents were determined barometrically during the gas extraction process, and dissolved concentrations of gas components (H_2_, CH_4_, CO_2_, etc.) were determined using an aliquot of subsamples using a gas chromatograph equipped with a pulse discharge detector (PDD). Major elements, including K, were determined by a Dionex ion chromatograph (ICS1500) after 200 times dilution.

### Sampling and preservation of animals

Hydrothermal vent megafauna were collected from the Dodo and Solitaire fields with permission from the Mauritius Prime Minister's Office, using a suction sampler attached to the HOV *Shinkai 6500*. The recovered animals were immediately preserved at −80°C for nucleic acid analyses and biochemical analyses, or fixed in 10% formalin-seawater and stored in 70–80% ethanol for subsequent morphological and anatomical analyses. Some were dissected and each of the organs was separately frozen at −80°C for subsequence nucleic acid analyses and biochemical analyses, or fixed in phosphate buffer saline (PBS) containing 5% (w/v) paraformaldehyde or 2.5% (w/v) glutaraldehyde and then stored in 70–80% ethanol or frozen at −80°C for microscopic analyses.

### Morphological observations

Shell sizes (width and height) of the ‘scaly-foot’ gastropods collected from the Solitaire (*N* = 139) and Kairei (*N* = 27) fields were measured and the differences in their proportions and sizes were examined. The shell width and shell height were positively linearly correlated (*R* = 0.9249, *P*<0.01) and therefore only the shell width was used to visualize the size distributions of the two populations. The average size of the two ‘scaly-foot’ populations was examined by Wilcoxon-Mann-Whitney test.

The morphological features were observed and the opercular plates and sclerites were carefully removed. The morphology of the opercula and the sclerites were observed under a compound microscope (Leica, MZ-3). The photographs were taken by Digital Sight DS-2MV (Nikon Co., Tokyo) through the ACT-2U application (Nikon Co., Tokyo).

### DNA extraction and sequencing

For DNA extraction from ‘scaly-foot’ gastropod specimens, we used the frozen black ‘scaly-foot’ morphotypes that were collected during cruise YK05–16 in 2006, and the frozen Solitaire morphotypes sampled during the present cruise. DNA extraction from the mantle tissues of these morphotypes was carried out using a DNeasy Tissue kit (QIAGEN, Valencia, CA) according to the manufacturer's protocol. The amplification of mitochondrial COI gene fragments was performed by PCR using a primer set of COI-6 [28] and LCO1490 [29] as previously described [30]. The obtained fragments were directly sequenced using an ABI3130 automated sequencer (Applied Biosystems). The obtained sequences were processed with the software package ATGC (GENETYX Co., Tokyo) to estimate a parsimonious haplotype network using the software package TCS v.1.21 [31], under conditions of 95% connection limit without gaps. In addition, a fragment of nuclear SSU rRNA gene sequence of each three individuals from the Solitaire and Kairei Fields was amplified with a universal primer set of 18e and 18dh [32] and sequenced after cloning, due to intra-individual polymorphism. The parsimonious networks were reconstructed based on a total of 40 sequences using the same methods as for the COI haplotypes. The list of the 40 sequences was shown as the phylogenetic tree in Figure S1.

### Microscopic and Scanning Electron Microscopy-Energy Dispersive X-ray Spectroscopy (SEM-EDS) analysis

Sclerites from the two morphotypes of ‘scaly-foot’ gastropods were detached from the foot and were embedded in epoxy resin. The long and short sections of each sclerite were then polished. The polished surface was observed using a VHX-900 digital microscope (KEYENCE, Osaka, Japan) and then subjected to platinum-deposition. SEM-EDS analyses were performed using a JSM-6500F (JEOL, Tokyo Japan) at an acceleration voltage of 15-kV.

### Comparative mechanical properties of sclerites

To evaluate the mechanical strength of sclerites from the two morphotypes of ‘scaly-foot’ gastropods, a three-point bending test along the long axis of the flat surface (according to the standards of Semiconductor Equipment and Materials International G86-0303) was performed by a Table-top universal testing instrument, EZGraph-10kN (Shimadzu, Kyoto, Japan). A wet sclerite was set onto two fulcrums, the clearance of which was 2 mm, and then the sclerite was pushed by a punch with a 1 mm/min velocity. To calculate stress (*σ*), the maximum power (*F*) until breaking of the sclerite was fitted into the following equation,
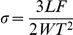
where *L*, *W*, or *T* signifies the span distance between fulcrums, the width of the sclerite, and the thickness of the sclerite, respectively.

### Nucleotide sequence accession numbers

The partial SSU rRNA gene and mitochondrial COI gene sequences from ‘scaly-foot’ gastropod morphotypes from the Kairei and Solitaire sites that were obtained in this study were assigned the accession numbers in DDBJ (DNA data bank of Japan) of AB540629 to AB540646, AB543244 to AB543246, AB634505 to AB634513, and AB691090 to AB691129.

## Supporting Information

Movie S1 **A movie showing the hydrothermal vents and associated faunas in the Dodo and Solitaire hydrothermal fields, recorded by HOV** ***Shinkai 6500*** **during the JAMSTEC cruise YK09–13.**
(M4V)

Figure S1 **The phylogenetic tree of the 40 sequences detected from each of the three individuals in the Solitaire and Kairei fields. The lattermost part of the labels of OTUs indicates identification number of clones from a single individual.**
(TIF)

Table S1 **Comparison of the presence of genera in hydrothermal vent fauna between the CIR and other regions, based on the report by Bachraty et al. (2009).**
(XLS)
