# Supplementary figures and images for: Discovery of New Hydrothermal Activity and Chemosynthetic Fauna on the Central Indian Ridge at 18°–20°S
Source: PLoS One. 2012 Mar 14;7(3):e32965. doi: 10.1371/journal.pone.0032965 (PMC3303786; doi:10.1371/journal.pone.0032965)

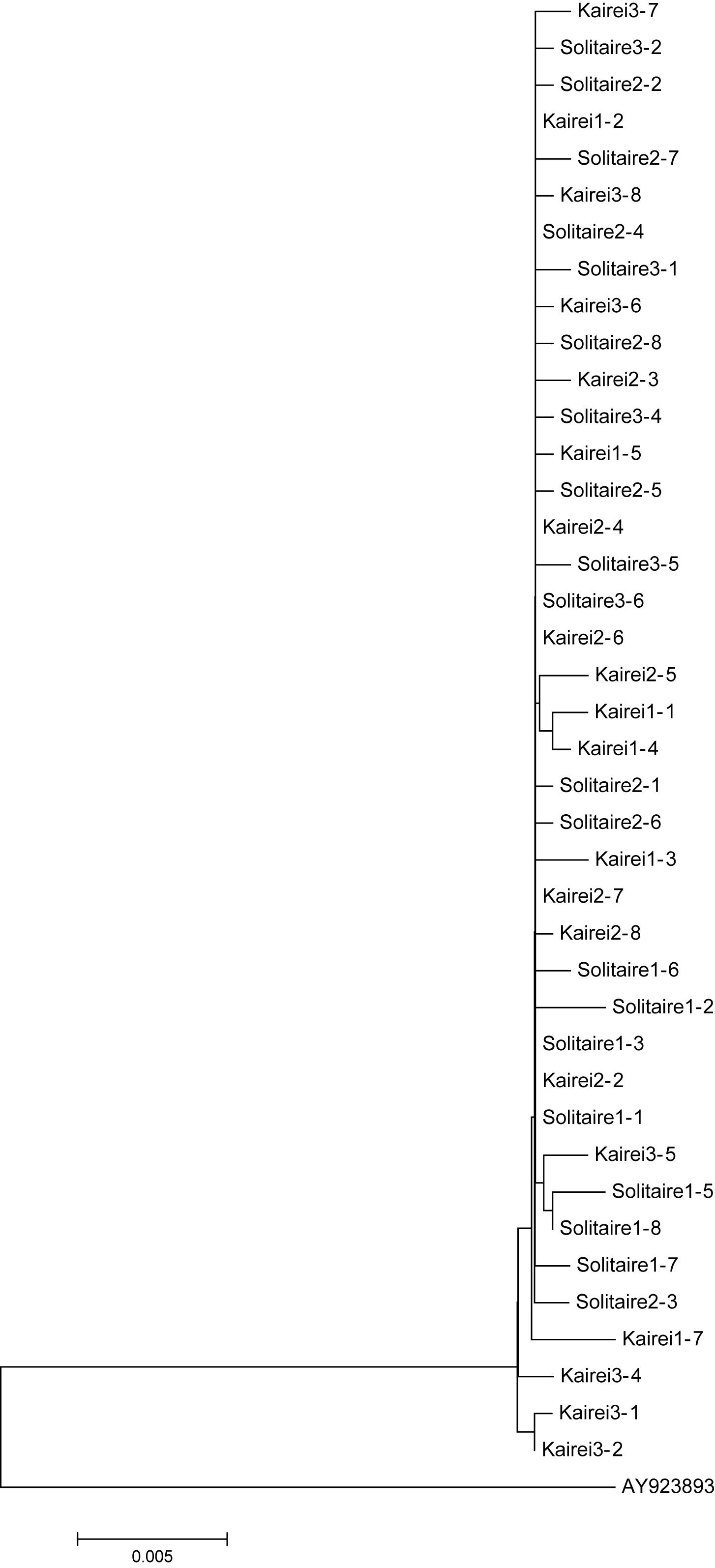

Supplement: Figure S1 — The phylogenetic tree of the 40 sequences detected from each of the three individuals in the Solitaire and Kairei fields. The lattermost part of the labels of OTUs indicates identification number of clones from a single individual. (TIF) [file pone.0032965.s002.tif]
